# Outcome of Acute Renal Injury in Diabetic Mice with Experimental Endotoxemia: Role of Hypoxia-Inducible Factor-1**α**

**DOI:** 10.1155/2013/254529

**Published:** 2013-07-31

**Authors:** A. Ortega, A. Fernández, M. I. Arenas, P. López-Luna, C. Muñóz-Moreno, I. Arribas, N. Olea, L. García-Bermejo, J. Lucio-Cazana, R. J. Bosch

**Affiliations:** ^1^Laboratory of Renal Physiology and Experimental Nephrology, Department of Biological Systems/Physiology Unit, University of Alcalá, Alcalá de Henares, Madrid, Spain; ^2^Department of Biological Systems/Physiology Unit, University of Alcalá, Alcalá de Henares, Madrid, Spain; ^3^Department of Clinical Chemistry University Hospital “Príncipe de Asturias”, University of Alcalá, Alcalá de Henares, Spain; ^4^Cell Response to Ischemia Laboratory, Department of Systems Disorders and Cancer, Instituto Ramón y Cajal de Investigación Sanitaria, Madrid, Spain

## Abstract

The role of diabetic nephropathy in the outcome of acute renal injury (AKI) is not well defined. Herein we evaluate the outcome of lipopolysaccharide- (LPS-) induced AKI in streptozotocin-induced diabetes, as well as the potential role of Hypoxia Inducible Factor (HIF-1**α**) in this condition. Although 6 h after LPS injection all mice developed a decrease in renal function, proteinuric diabetic mice showed a better recovery of this parameter throughout the study (72 h). Both HIF-1**α** and vascular endothelium growth factor (VEGF) were found to be upregulated in diabetic mice. After LPS injection, all animals showed an upregulation of these factors, although it was higher in the diabetic group. Glycated albumin (GA) was found to upregulate HIF-1**α** in HK-2 cells, which resulted in increased production of VEGF. Interestingly, LPS cooperated with GA to induce HIF-1**α** upregulation. In conclusion, diabetic mice display a better recovery of AKI after experimental endotoxemia. Moreover, these animals showed an increased expression of both HIF-1**α** and VEGF that was reproduced by incubating renal cells with GA. Since VEGF is considered a survival factor for tubular cells, our findings suggest that diabetes displays HIF-1**α** upregulation that might function as a “precondition state” offering protection from endotoxic AKI.

## 1. Introduction

The role of diabetes in the outcome of acute renal injury (AKI) is not well understood and may depend upon the cause of the injury as well as on the stage of diabetic renal involvement. Among the various causes of AKI, endotoxemia, a major component of sepsis, remains an elusive and challenging condition which is still lacking treatment. Although it is known that rodents with experimental diabetes are protected from certain nephrotoxic agents [1–3], diabetes has been recognized as an independent risk factor for the development of AKI in a variety of clinical settings, including sepsis [4–8]. 

 Hypoxia-inducible factor (HIF-1*α*) allows the adaptive response to hypoxia by stimulating the expression of target genes such as erythropoietin, enzymes involved in glucose metabolism, and the vascular endothelial growth factor A (VEGF-A) [9]. The later has been recognized as a survival factor for proximal tubular cells [10].

 HIF-1*α* is a heterodimer transcription factor consisting of a constitutively expressed *β* subunit and two *α* subunits, HIF-1*α* or HIF-2*α*. In normoxia, HIF-1*α* is continuously synthesized but rapidly ubiquitinated and subsequently degraded by the cellular proteasome [11]. Under hypoxia, the HIF-1*α* ubiquitination is suppressed. HIF-1*α* protein is thereby stabilized, it translocates to the nucleus, and together with the *β* subunit and transcriptional coactivators, it binds to hypoxia-responsive elements (HRE) in target genes [12, 13]. Besides hypoxia, several physiological regulators such as growth factors, hormones, stress factors, and inflammatory mediators, increase HIF-1*α* expression in normoxia [14]. Moreover, HIF-1*α* is also induced upon diabetic condition with a potential role in wound healing [15]. Activation of HIF-1*α* by cobalt chloride has therapeutic benefit in several kidney disease models including ischemia reperfusion, cisplatin nephropathy, remnant kidney, progressive anti-Thy1 nephritis, and diabetic nephropathy, reviewed by Nangaku et al. [16]. Less is known about the potential role of HIF-1*α* in AKI due to endotoxemia. Moreover, most of the available data are based upon experimental models of early diabetic stage, which might not recapitulate a long-term disease such as diabetic nephropathy, a condition characterized by an increase in urinary albumin excretion (UAE) [17]. There is also tubular injury, which is due to several factors, particularly high glucose levels, albuminuria, and the presence of advanced glycation end-product (AGE-) modified proteins [17, 18].

The aim of the present study was to evaluate the outcome of AKI in animals with experimental diabetes after the development of an increase in the UAE. Moreover, we also investigate the potential role of HIF-1*α* in this outcome.

## 2. Material and Methods

In all of the experiments, adult CD-1 mice (4–8 months old, *n* = 10–15 per group, total 104) were used. The experimental procedures were previously approved by the Committee for Animal Ethics of Alcalá University, in accordance with the Spanish and European Guidelines. AKI was induced by intraperitoneal injection of lipopolysaccharide (LPS) (10 mg/Kg) (from *E. Coli*, Sigma) in mice at different periods (6–72 h). In some animals, diabetes was induced by three consecutive daily intraperitoneal injections of streptozotocin (STZ) (Sigma, St. Louis, MO, USA), 65 mg/kg body weight in citrate buffer, pH 4.5 (vehicle) 6 weeks before LPS injury. This is a previously reported model of early diabetic nephropathy characterized by increased UAE during the first month of diabetes [19]. After the last STZ injection, induction of diabetes was confirmed by measurement of blood glucose levels. Animals with blood glucose >300 mg/dL were included in the study.

Animals were individually housed in metabolic cages with free access to food and tap water, and 24-hour urine was collected for protein measurement. Blood was taken by cardiac puncture under ether anaesthesia, and plasma glucose was determined [19]. Urinary albumin excretion (UAE), endogenous creatinine clearance (CCr), urinary volume (UV), and fractional excretion of sodium (NaFE) (Na_*u*_ × Cr_*s*_)]/[Na_*s*_ × Cr_*u*_] × 100) were analyzed. One kidney of each animal was removed, weighed, frozen in liquid nitrogen, and stored at −80°C for subsequent total protein and RNA extraction. The remaining kidney was fixed in 10% (v/v) formaldehyde in PBS, embedded in paraffin, and sectioned at 5 *μ*m for morphological and immunohistochemistry studies. 

### 2.1. Renal Expression and Immunolocalization of HIF-1*α* and VEGF-A

Western blotting in tissue was performed as previously described [20]. Briefly, a small piece of kidney was lysed in RIPA buffer and centrifuged for preclearance, and total protein was loaded into 8% acrylamide SDS gels and transferred to PVDF membrane. The membrane was incubated with anti-mouse anti-HIF-1*α* (R&D Systems, Inc, MN, USA), 1/500 and appropriate HRP-conjugated secondary antibody. 

Sections were deparaffinised, rehydrated, and placed in 10 mM sodium citrate buffer, pH 6.0, and heated in a pressure cooker for 2 min. The sections were allowed to cool for 20 min. After rinsing with distilled water, the sections were washed twice in TBS buffer, pH 7.6, for 5 min. The endogenous peroxidase activity was inhibited by incubation with 3% H_2_O_2_ for 20 min. Sections were washed with H_2_O and TBS and incubated with 3% normal donkey serum plus 0.05% Triton X-100 in TBS, pH 7.6, at room temperature for 45 min, to prevent nonspecific binding of the first antibody. Afterwards, the sections were incubated overnight at 4°C, with the following rabbit polyclonal antibodies: HIF-1*α* (Abcam, Cambridge, UK) diluted 1 : 300 and VEGF-A (Santa Cruz Biotechnology, Lamecula, CA, USA) diluted 1 : 500 in the blocking solution diluted 1 : 9. Then, the sections were washed in TBS, and detection was made by the conventional labelled-streptavidin-biotin method (LSAB-kit, Dako). The peroxidase activity was detected using the DAB kit (Master Diagnostica, Granada, Spain). Tissue sections were counterstained with hematoxylin, dehydrated, cleared in xylene, and mounted in Entellan (Merck, Darmstadt, Germany). Two independent observers in a blinded manner scored both HIF-1*α* and VEGF-A renal staining as negative, mild, moderate, and intense. The final score was the mean of the two evaluations.

### 2.2. Cell Culture

Human kidney HK-2 cells were purchased from American Type Culture Collection (Rockville, MD, USA). YC-1 was purchased from Sigma Chemical Co. (St. Louis, MO, USA), monoclonal HIF-1*α* (1 : 1000) (Transduction Laboratories, BD Biosciences, Palo Alto, CA, USA); polyclonal *β*-actin (1 : 10000) was obtained from Sigma. Cells were maintained in DMEM supplemented with 10% foetal bovine serum (FBS), 1% penicillin/streptomycin/amphotericin B (Invitrogen, Carlsbad, CA, USA) and 1% Insulin-Transferrin-Selenium (Sigma, St. Louis, MO, USA). Cells were routinely cultured in 95% air, 5% CO_2_ (normoxic conditions) at 37°C. In all experiments, cells were plated at 70–90% confluence, and when completely attached, they were treated with LPS in serum complete media. When necessary, cells were also treated with AGE, albumin, or YC-1 under the conditions specified in the legends to figures. 

### 2.3. Single-Step Real-Time Quantitative RT-PCR

Total cell RNA from HK-2 cells was isolated with TriReagent (Sigma, St. Louis, MO, USA), and real-time quantitative RT-PCR analysis was performed in 2 ng samples using SYBR Green PCR master mix (Applied Biosystems), in one-step RT-PCR protocol as previously described [20]. Primer sequences for genes were as follows (sequences 5′-3′): VEGF165 sense: GACAAGAAAATCCCTGTGGGCAAC, antisense: GCGAGTCTGTGTTTTTGC; *β*-actin sense: AGAAGGATTCCTATGTGGGCG and antisense: CATGTC CCAGTTGGTGAC.

### 2.4. Protein Isolation and Western Blotting

HK-2 cells were incubated with albumin or AGE (100 *μ*g/mL each) or in combination with LPS (1 *μ*g/mL) for different periods. Cells were washed twice with ice-cold PBS, then harvested, scraped into ice-cold PBS, and then pelleted by centrifugation at 500 ×g for 5 min at 4°C. In order to obtain cell lysates, the cells were kept on ice for 30 min in a solution containing 50 mM Tris-HCl (pH 7.5), 150 mM NaCl, 1% Triton X-100, 0.5% sodium deoxycholate, and protease inhibitors. Thereafter, the cells were pelleted by centrifugation at 4000 ×g for 5 min at 4°C. Proteins from cell lysates were denatured by heating. Then, they were resolved by 10% SDS-PAGE and blotted on a nitrocellulose membrane (BioTrace/NT) overnight in 50 mM Tris-HCl, 380 mM glycin, 0.1% SDS, and 20% methanol. Mouse anti-HIF-1*α* (1 : 1000) antibody (BD Biosciences, CA, USA) was added followed by incubation overnight at 4°C. After treatment for 1 h at room temperature with the corresponding secondary antiserum (1 : 4000), the signals were detected with enhanced chemiluminescence reagent (GE Healthcare-Life Science, NJ, USA) using *β*-actin antibody (Calbiochem-Merk Bioscience, USA) as loading control. 

For HIF-1*α* inhibition, we used HIF-1*α* siRNA sc-44225 (Santa Cruz Biotechnologies, CA, USA) for HIF-1*α* containing 3 sequences against 3 different HIF-1*α* exons and scramble siRNA AM4637 (Ambion) as a control as previously described [16]. HK-2 cells at 70% of confluence were transfected with 100 nM HIF-1*α* siRNA or 100 nM scramble siRNA. According to the manufacturer's protocol, we used Lipofectamine 2000 to get the transfection. HIF-1*α* interference was evaluated by qRT-PCR for HIF-1*α* mRNA. 

### 2.5. Statistical Analysis

 Results are expressed as mean ± SEM throughout the text. Animal data was analyzed by either the Kruskal-Wallis test or Mann-Whitney test, when appropriate. Unless otherwise specified, in vitro experiments were repeated at least three times, and the statistical analysis was performed by the Bonferroni test. In all cases, a *P* < 0.05 was considered statistically significant.

## 3. Results

### 3.1. Characterization of LPS-Induced AKI in Diabetic Mice Model

**Figure 1:**
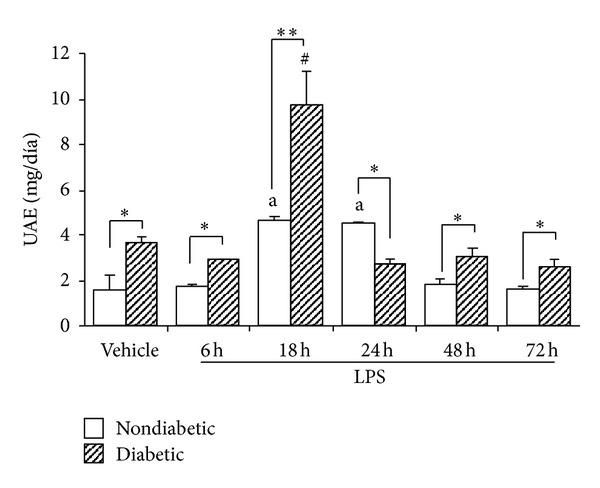
Urinary Albumin Excretion (UAE) in diabetic and nondiabetic LPS-injected mice. Values are mean ± SEM. *P < 0.05 versus control value, **P < 0.01 versus control value, ^#^ *P* < 0.01 versus diabetic, and a *P* < 0.01 versus nondiabetic.

**Figure 2:**
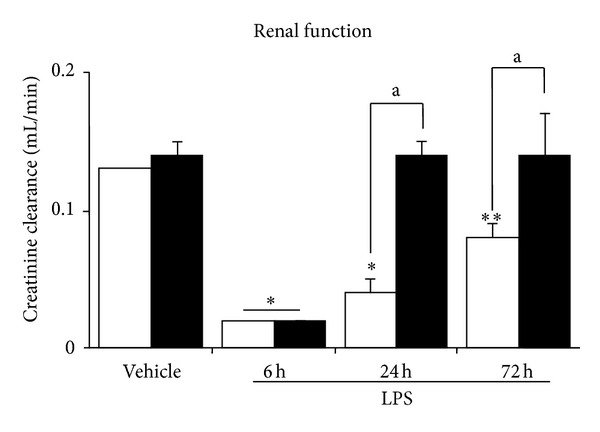
Renal function (endogenous creatinine clearance (CCr) in diabetic (black bars) and nondiabetic (open bars) LPS-injected mice. Values are mean ± SEM. *P < 0.05 versus control value, **P < 0.01 versus control value, and a *P* < 0.01 versus nondiabetic.

**Figure 3:**
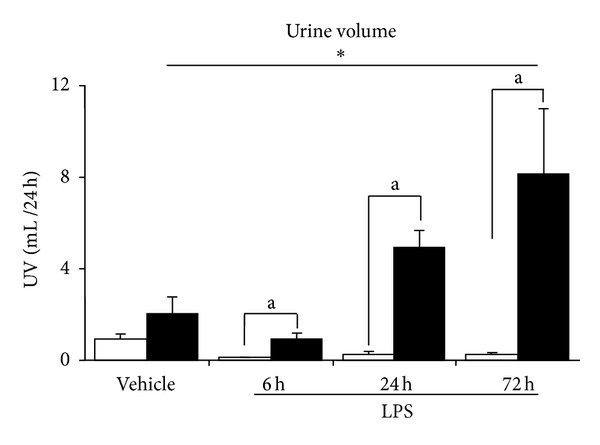
Urinary volume in diabetic (black bars) and nondiabetic (open bars) LPS-injected mice. Values are mean ± SEM. *P < 0.05 versus control value and a *P* < 0.01 versus nondiabetic.

**Figure 4:**
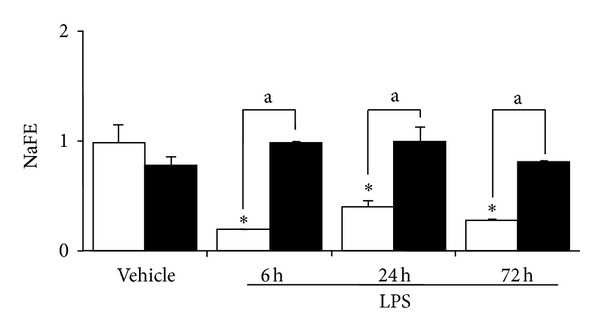
Fractional excretion of sodium (NaFE) in diabetic (black bars) and nondiabetic (open bars) LPS-injected mice. Values are mean ± SEM. *P < 0.05 versus control value and a *P* < 0.01 versus nondiabetic.

As shown in Figure 1, diabetic animals after 6 weeks of STZ display a significant increase in the UAE throughout the study. Both groups of animals also showed a significant increase of this parameter at 18–24 h after LPS injection. Although six hour after LPS, diabetic and nondiabetic mice developed a similar degree of decrease of renal function, only diabetic animals showed a recovery of this parameter at 24 h to a level not significantly different from those of the control animals (Figure 2). On the contrary, nondiabetic mice did not show a complete recovery of the renal function throughout the period of the study (72 h) (Figure 2). As expected, these changes were associated with an increased Urine Volume (UV) in diabetic mice, and a decrease in the UV in nondiabetic mice (Figure 3). Moreover, while diabetic mice did not show any significant changes in the NaFE throughout the study, nondiabetic mice displayed a significant decrease in this parameter (Figure 4). This maintained renal sodium reabsorption capacity observed in the diabetic mice is in accordance with a functional or mild form of AKI [21]. 

### 3.2. Renal Expression and Immunolocalization of HIF-1*α* and VEGF-A

**Figure 5:**
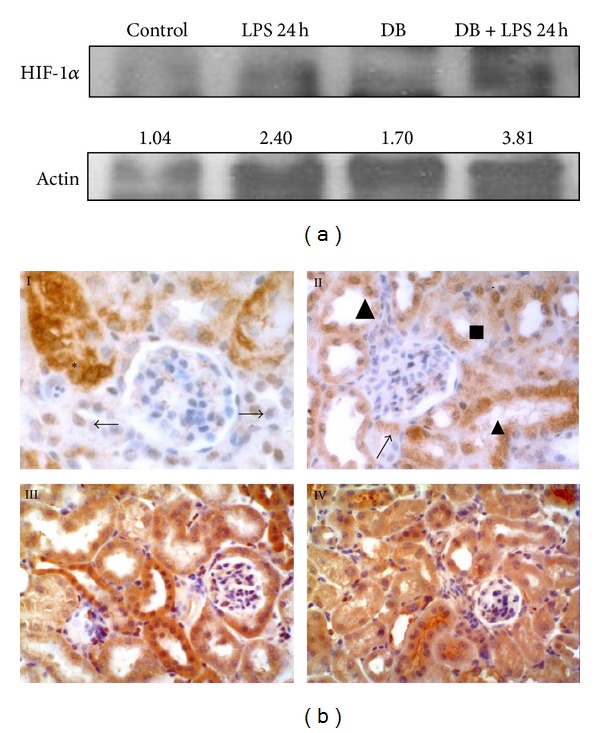
(a) Western-blotting analysis of HIF-1*α* in renal tissue. Quantification of HIF-1 *α* signal normalized by *β*-actin signal is also shown. (b) HIF-1*α* immunohistochemistry. I: control kidney. An intense immunoexpression of HIF-1*α* was detected in the cytoplasm of proximal convoluted tubules (∗), and in the nuclei of distal tubules, the labelling was weaker (→). II: diabetic mice. Proximal tubules showed positive reaction to HIF-1*α* in the cytoplasm (■). Both nucleus and cytoplasm of distal convoluted tubules cells (▲) presented a positive reaction to HIF-1*α*. The arrow points to the urinary pole with moderate reaction to this antibody. III: LPS-treated mice. Both convoluted tubules showed a strong immunolabelling to HIF-1*α*. IV: diabetic LPS-treated mice. The highest reaction to HIF-1*α* was encountered in the kidney of diabetic mice treated with endotoxin. Magnification: I and II: ×600; III and IV: ×300.

Western-blotting analysis revels that LPS increased the expression of HIF-1*α* in both groups of animals, albeit it was even higher in the diabetic group (Figure 5(a)). Interestingly, the condition of diabetes itself was able to induce a significant upregulation of this protein. 

In the kidney of control animals, an intense immunoexpression to HIF-1*α* was observed in the cytoplasm and several nuclei of the proximal convoluted tubules, and a weak signal was observed in the nuclei of distal tubules (Figure 5(b), I). By contrast, 5 weeks after diabetes induction, the mouse kidney displayed a higher number of positive tubuloepithelial nuclei, showing an increase in the cytoplasm immunoreactivity (Figure 5(b), II). 

In LPS-treated mice, both the nucleus and cytoplasm of renal tubules showed a strong immunoexpression to HIF-1*α* antibody (Figure 5(b), III) and this labelling was even greater in diabetic LPS-treated mice (Figure 5(b), IV).

**Figure 6:**
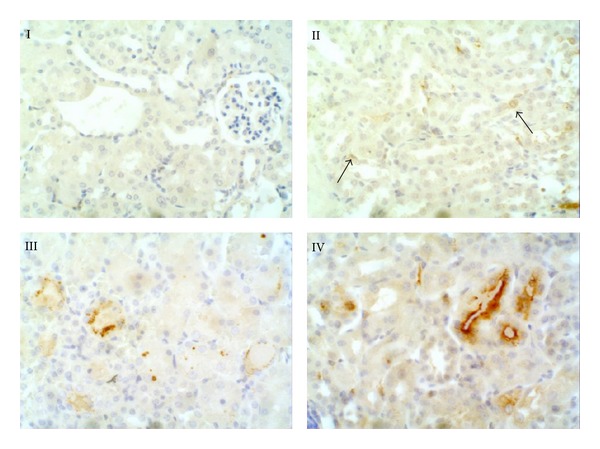
VEGF-A immunohistochemistry. I: control kidney. VEGF-A was not detected in the kidney of control mice. II: diabetic mice. Some of proximal tubules cells showed positive reaction to VEGF-A in the cytoplasm (right arrow). Endothelial cells were also immunostained (left arrow). III: LPS-treated mice. In some cells of convoluted tubules, VEGF-A was located in the cytoplasm with a granular pattern; in the other tubules, VEGF-A antibody showed a diffuse labelling. IV: diabetic LPS-treated mice. The highest reaction to VEGF-A was encountered in the kidney of diabetic mice treated with endotoxin. Magnification ×300.

To analyze if the observed HIF-1*α* upregulation was transcriptionally active in vivo, we analyzed the expression of the HIF-1*α* target gene VEGF-A, by immunostaining in mice kidneys. We first observed that while control mice did not show VEGF-A staining, diabetic mice display some degree of expression of this protein. Although all LPS-treated mice display a significant increase in the expression of VEGF-A in comparison with their respective controls, diabetic LPS-treated mice showed the highest immunolabeling (Figure 6).

### 3.3. Glycated Albumin Synergizes with LPS to Upregulate HIF-1*α* in HK-2 Renal Cells

**Figure 7:**
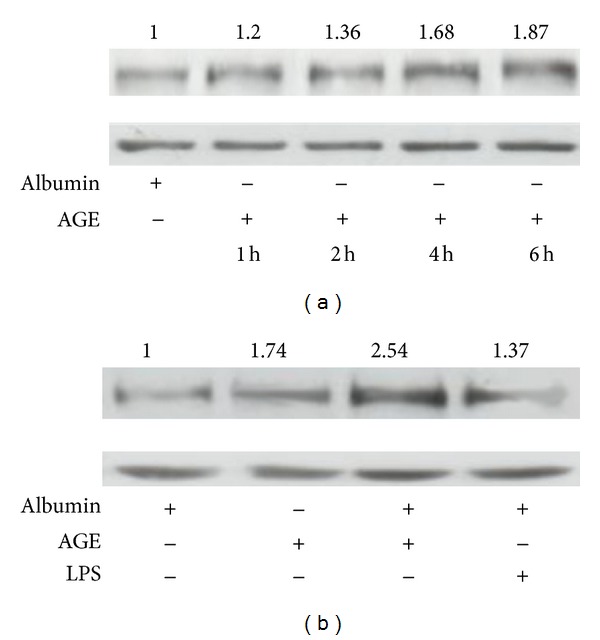
AGE and LPS induce HIF-1*α* upregulation and cooperate to over-increase HIF-1*α* expression. (a) AGE induces HIF-1*α* upregulation. (b) LPS induces HIF-1*α* upregulation and synergizes with AGE to over-increase HIF-1*α* expression. Equal protein loading was confirmed by probing with an anti-*β*-actin antibody. Normalized density ratio of HIF-1*α* over *β*-actin is indicated for each band.

We then performed an in vitro approach in order to get inside into the cellular mechanism responsible for the observed upregulation of both HIF-1*α* and VEGF-A in the kidney of diabetic mice. It has been previously described that AGE increases VEGF expression in retinal epithelial cells through activation of HIF-1*α* [22]. AGE is the major form of circulating glycated proteins in vivo, and its levels are increased in diabetes [23, 24]. Furthermore, AGE elicits pathobiological effects in cultured renal cells that are identical to those of high glucose ambient [23]. Therefore, in order to assess the effect of the diabetic milieu on the expression of HIF-1*α* in proximal tubular cells, HK-2 cells were incubated with 100 *μ*g/mL AGE or albumin for up to 6 h. In these conditions, HIF-1*α* was upregulated in a time-dependent manner in AGE-treated cells, but not in cells treated with albumin, and its expression reached its maximum after 4 h incubation (Figure 7(a)). 

We then assessed whether LPS affects AGE-induced HIF-1*α* upregulation, since a hypothetical synergy for HIF-1*α* expression in the proximal tubule could contribute to better recovery of renal function in diabetic mice after experimental endotoxemia. HK-2 cells were preincubated with either 100 *μ*g/mL AGE or 100 *μ*g/mL albumin for 1 h and then with 1 *μ*g/mL LPS for 5 h. Expression of HIF-1*α* was increased to a similar degree by AGE and LPS, but it was dramatically enhanced in cells which were incubated with both agents (Figure 7(b)). These results suggest that AGE and LPS increased HIF-1*α* expression in the proximal tubules, in a synergistic manner.

### 3.4. Glycated Albumin-Induced HIF-1*α* Promotes the Upregulation of the Renoprotective VEGF-A in HK-2 Renal Cells

**Figure 8:**
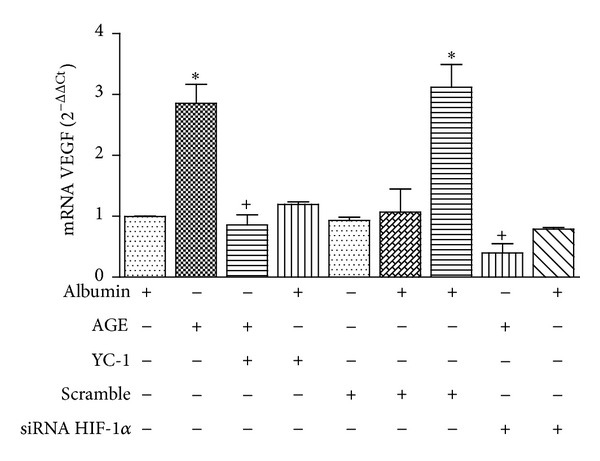
AGE increases VEGF mRNA expression in a HIF-1*α*-dependent manner. Cells were preincubated with HIF-1*α* inhibitor YC-1 or transfected with HIF-1*α* siRNA or control siRNA (scramble) as indicated in Section 2. Then they were incubated with albumin or AGE, and VEGF mRNA expression was quantified by Q-RT-PCR. Bars are the mean ± SD of 3 different experiments. *P < 0.01 versus albumin; ^+^ *P* < 0.01 versus AGE.

To demonstrate that AGE-induced HIF-1*α* was transcriptionally active, we determined by Q-RT-PCR the mRNA levels of VEGF-A in HK-2 cells treated with either 100 *μ*g/mL GA or 100 *μ*g/mL albumin for 5 h. VEGF mRNA expression (Figure 8) was substantially increased by AGE but not by albumin. In order to establish the dependency on HIF-1*α* of AGE-induced increase in VEGF-A mRNA expression, cells were preincubated with HIF-1*α* inhibitor YC-1 or transfected with specific HIF-1*α* siRNA. Both treatments abolished AGE-induced increase in VEGF-A mRNA expression (Figure 8), which confirmed that the AGE effect on VEGF-A expression is dependent on HIF-1*α*.

## 4. Discussion

Although it is known that rodents with experimental diabetes are protected from certain nephrotoxic agents [1–3], most of the available data are based upon experimental models of an early diabetic stage, condition characterized by the presence of renal cell proliferation, and thus renal cells are not quiescent as occurred in adult subjects [25, 26]. Thus, as reported by Zhang et al. [25], an inherently proproliferative state of the diabetic kidney may explain the observed lower expansion of renal injury on the one hand and recovery due to higher proliferative response after injury on the other. The same also accounts for HIF-1*α* where available data is limited to an early diabetic stage. Thus, herein we studied the role of HIF-1*α* in the outcome of AKI in diabetic animals after the development of proteinuria.

AKI is a syndrome defined by an abrupt change in the renal function and/or urine output which could range from overt tubular necrosis to mild perturbations in renal function without significant pathologic changes (prerenal azotemia) [18]. In our study, we observed that diabetic mice, besides an increase in the UAE, display better recovery of renal function after experimental endotoxemia. The maintained renal sodium reabsorption capacity observed in the diabetic mice is in accordance with a functional or mild form of AKI [21]. Furthermore, no alterations of the renal structure in any of the animal groups studied were found. 

This is interesting due to the fact that proteinuria is a well-known factor involved in the progression of renal damage by mechanisms which include alterations in tubuloepithelial cell growth, apoptosis, gene transcription, and inflammatory cytokine production [27]. Furthermore, multiple clinical trials have indicated that antiproteinuric strategies are, in general, renoprotective [27]. Herein we demonstrate that in the diabetic kidney, endotoxemia could trigger the upregulation of HIF1-1*α* even in the proteinuric stage of the disease.

 It is known that renal proximal tubular epithelial cells are targets for LPS during sepsis and renal infections. Septic AKI is characterized by a paucity of tubular cell death despite often severe impairment of global function. Accumulating data suggest that the renal tubules are also heavily involved in the pathogenesis of diabetic nephropathy [16]. In diabetes, tubular injury in the kidney is due to several factors, particularly high glucose levels, albuminuria, and the presence of advanced glycation end-product modified proteins. We show here that either LPS or AGE increases the expression of HIF-1*α* in HK-2 cells to a similar extent, and that exposure of AGE-treated cells to LPS results in overexpression of HIF-1*α*. Interestingly, renal expression of HIF-1*α* was also increased in several areas, including proximal tubules, in both diabetic and LPS-treated mice, and maximal renal HIF-1*α* upregulation was found in diabetic mice treated with LPS. 

 Accumulating evidence indicates that there is a fine line between the potential benefit and harmful side effects of HIF-1*α* activation. For instance, among the noxious effects of HIF-1*α*, which is related to our experimental setting, it is worth mentioning that hypoxia-dependent increased tubular activity of HIF-1*α* has been proposed as a contributing factor in the development of tubulointerstitial fibrosis in diabetes mellitus [16]. In addition, HIF-1*α* has been shown to play an essential role in the development of LPS-induced sepsis in mice, so that targeted deletion of HIF-1*α* has a protective effect [28]. On the other hand, HIF-1*α* inducer cobalt chloride improves disease manifestations in a variety of kidney disease models including diabetic nephropathy and hypoxic preconditioning ameliorates LPS-induced renal dysfunction [29]. Moreover, it has been recently published that HIF-1*α* promotes renal ischemic injury regeneration [30]. According to the latter observation, it is tempting to speculate that the increased renal expression of HIF-1*α* in diabetic mice, particularly in the proximal tubules, might contribute to better recovery of renal function after experimental endotoxemia. 

Regarding potential mechanisms triggered by HIF-1*α* for contributing to renal recovery in our model, it is conceivable that VEGF induction as HIF-1*α* target gene might be mediating. VEGF has been described as a survival factor for proximal tubule cells [23] and critical for maintenance of renal vasculature during kidney damage including AKI [31]. Our present data indicating that VEGF is induced in LPS-treated diabetic mice further support this notion.

In conclusion, we have found that mice with diabetic nephropathy display better recovery of renal function after experimental endotoxemia than their control littermates. This finding was associated with an increased expression of both HIF-1*α* and VEGF-A in the tubule epithelium of the diabetic mice. Furthermore, in these cells, AGE was found to be capable of upregulating HIF-1*α* as well as VEGF-A, a survival factor for these cells. Our findings suggest that even in the presence of an increased UAE, the diabetic condition may display upregulation of HIF-1*α* that might function as a “precondition state” capable of protecting from renal damage such as endotoxic AKI.
